# Acaricidal activity of small molecule antagonists of the tick kinin receptor against *Rhipicephalus microplus* acaricide‐susceptible and resistant strains

**DOI:** 10.1002/ps.70309

**Published:** 2025-10-25

**Authors:** Waleed M. Arafa, Kimberly H. Lohmeyer, Patricia V. Pietrantonio

**Affiliations:** ^1^ Department of Entomology Texas A&M University College Station Texas USA; ^2^ Knipling‐Bushland U.S. Livestock Insects Research Laboratory USDA‐ARS Kerrville Texas USA; ^3^ Present address: Parasitology Department, Faculty of Veterinary Medicine Beni‐Suef University Beni‐Suef Egypt

**Keywords:** cattle fever tick, probit analysis, kinin receptor antagonists, G protein‐coupled receptor, larval and adult immersion test, methylated rapeseed oil, DMSO

## Abstract

**BACKGROUND:**

Resistance to acaricides underscores the need for tick control alternatives. We previously identified in a high‐throughput screen six small molecules SACC‐0412060, SACC‐0412062, SACC‐0412066, SACC‐0064443, SACC‐0428788, and SACC‐0039590 as antagonists of the kinin receptor from *Rhipicephalus microplus*. The acaricidal activity of these molecules was investigated.

**RESULTS:**

The toxicities of adjuvant/solvent combinations of a rapeseed oil methyl ester or a propylene carbonate in 5% DMSO *versus* 5% DMSO alone were evaluated using a larval immersion test (LIT) with the *R. microplus* susceptible Deutch strain, and all were non‐toxic. The rapeseed oil methyl ester (1%) in 5% DMSO was adopted as the adjuvant for all molecules in bioassays. Permethrin (3.19 mM) killed all larvae. The Deutch strain mortality with SACC‐0039590 and SACC‐0428788 (both at 1 mM) was 100% and 73.65%, respectively, while ranged from 2.7% to 3.8% with the other small molecules. In the LIT, the LC_50_ of SACC‐0039590 and SACC‐0428788 were 60 μM, and 450 μM, respectively. For the *R. microplus* Arauquita pyrethroid‐resistant strain in the LIT, the LC_50_ of SACC‐0039590 and SACC‐0428788 were 60 μM and 237.5 μM respectively. SACC‐0039590 (N‐[2‐(4‐chlorophenyl)ethyl]‐5‐methylthieno[2,3‐d]pyrimidin‐4‐amine) and SACC‐0428788 (N‐[2‐(4‐chlorophenyl)ethyl]thieno[2,3‐d]pyrimidin‐4‐amine) are structural analogs, the first one featuring a 5‐methyl group absent in the second. For engorged Deutch females, immersion in SACC‐0039590 caused dose‐dependent reductions in egg mass weight (~35%) and egg hatching (21%–38.59%). Permethrin (3.19 mM) significantly reduced egg mass weight but not egg hatching.

**CONCLUSION:**

The small molecule SACC‐0039590 killed susceptible and pyrethroid‐resistant larvae of *R. microplus* and reduced the reproductive output of females. © 2025 The Author(s). *Pest Management Science* published by John Wiley & Sons Ltd on behalf of Society of Chemical Industry.

## Graphical abstract

Two small molecules, SACC‐0039590 and SACC‐0428788, killed cattle fever tick, *R. microplus*, susceptible Deutch strain and pyrethroid resistant Arauquita strain larvae. SACC‐0039590 at 1 mM decreased the reproductive parameters of Deutch females.

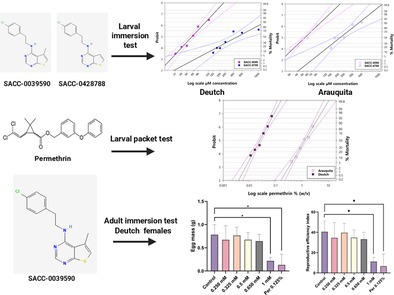

## 1 INTRODUCTION

In tropical and subtropical regions worldwide, the *Rhipicephalus microplus* tick known as cattle fever tick, Asian blue tick, or southern cattle tick in the USA, is the predominant blood feeding ectoparasite that causes severe economic losses for livestock and infests wildlife.^1^ It transmits the pathogens *Babesia bigemina, B. bovis*, and *Anaplasma marginale*, which threaten cattle survival,^2^, ^3^, ^4^ and reduce livestock productivity.^2^, ^5^ Although *R. microplus* has been eradicated from the USA since 1943, the USA‐Mexico border represents a constant risk for the reintroduction of the cattle fever tick, especially through the movement of wild animals.^6^, ^7^ Chemical acaricides are commonly used to control ticks on infested livestock. Unfortunately, acaricide resistance has emerged with the massive use of these chemicals.^8^ Globally, synthetic pyrethroid (SP) resistance was recorded in ticks from many countries, including the US, Australia, Brazil, Colombia, Mexico, India, and Egypt.^9^, ^10^, ^11^, ^12^, ^13^, ^14^, ^15^ Furthermore, double (SP and Organophosphates (OP), triple (SP, OP, and amidines (Am), and multi‐resistant (SP, OP, Am and ivermectin) *R. microplus* strains were reported.^16^, ^17^ Therefore, there is an urgent need for new alternative compounds that can control ticks.^18^

G protein‐coupled receptors (GPCRs) are cell membrane receptors that have seven α‐helical transmembrane regions. These receptors transduce extracellular signals and regulate intracellular second messengers. Because of the critical role of GPCRs in animal physiology, they are important drug targets for human diseases and candidates for tick control.^19^, ^20^ In *R. microplus*, the metabolite of the commercial acaricide amitraz, activates the GPCR for tyramine (previously thought as being an octopamine receptor),^21^ and amitraz resistance has been hypothesized to be mediated by a putative *R. microplus* β‐adrenergic‐like octopamine receptor.^21^ The IRAC mode of action classification 2025 lists amitraz as the only representative of group 19 as octopamine receptor agonist (https://irac-online.org/documents/moa-classification). Altogether, these examples provide proof‐of‐principle that targeting tick GPCRs could lead to acaricides with unique modes of action.^20^, ^22^, ^23^ Invertebrate neuropeptide GPCRs are often pleiotropic. The kinin receptor from *R. microplus* was the first GPCR discovered in the Acari, and it is transcribed in all life stages and both sexes of ticks.^24^ Insect kinins (IK) are neuropeptides that regulate vital biological functions in insects,^20^, ^25^ but less is known in ticks.^26^ Several studies showed that the kinin receptor is involved in myotropic and diuretic activities in insects,^27^, ^28^ and regulates meal size, feeding, starvation tolerance, digestive hormone release, and reproductive capabilities in arthropods and other invertebrates.^29^, ^30^, ^31^, ^32^, ^33^ Silencing the tick kinin receptor in females of *R. microplus* decreased larval hatching, the weight of egg masses, and the reproductive efficiency index.^29^ It was recently reported that in the tick *R. sanguineus* the kinin receptor is expressed in midgut muscles and activation by a tick kinin triggers peristalsis.^34^

The first aim of this study was to select a non‐toxic solvent and/or solvent/adjuvant combination that may aid the cuticular penetration of candidate small synthetic molecules tested for acaricidal activity. Subsequently, using this non‐toxic combination we evaluated six small synthetic molecules previously identified as kinin receptor antagonists in a high‐throughput screen or identified *in silico* as their structural analogs.^35^ The small molecules identified in the screen were also validated on the cell line BMLK3 expressing the recombinant *R*. microplus kinin receptor, exhibiting dose‐dependent antagonism.^35^ We assessed for *R. microplus* tick larvae and engorged females the acaricidal activity of the small molecules SACC‐0412066, SACC‐0412062, SACC‐0412060, SACC‐0064443 that share the same backbone but feature different aromatic groups,^35^ as well as the activity of SACC‐0039590 and its very similar structural analog SACC‐0428788. Additionally, effects on female reproductive output were evaluated.

## 2 MATERIALS AND METHODS

### 2.1 Ticks

Bioassays with *Rhipicephalus microplus* were conducted at the USDA‐ARS Cattle Fever Tick Research Laboratory (CFTRL) in Edinburg, Texas. Two *R. microplus* tick strains were utilized for bioassays. The Deutch acaricide‐susceptible reference tick strain, initiated from ticks collected in Laredo, Texas, has been maintained at the USDA‐ARS Cattle Fever Tick Research Laboratory (CFTRL) since 2001. The *R. microplus* Arauquita strain is resistant to pyrethroids and organophosphates, and originated from a ranch in Arauca State, Colombia,^15^ and is maintained at the CFTRL since 2018. Tick *R. microplus* research is approved under Animal Use Protocol TAMU AUP 2022‐0141.

### 2.2 Carriers and control treatments

Dimethylsulfoxide (DMSO, D‐2650, Sigma‐Aldrich, St Louis, MO) was prepared in deionized water (V/V). The lack of toxicity of DMSO 5% to *R. microplus* larvae and engorged female ticks was previously reported.^36^, ^37^ MERO® is a commercial wetting agent produced by Bayer® (Reading, UK) and contains 81.4% w/w oil (rapeseed fatty acid esters) (EAC1) and ethoxy (7) tridecanol. JEFFSOL® AG 1555 Carbonate (Huntsman Co., TX, USA) (hereafter JEFFSOL) is a solvent approved for agricultural pesticide formulations.^38^ MERO® at 1% (the label rate) in 5% DMSO (final DMSO concentration 4.95%) or 10% JEFFSOL in 5% DMSO (final DMSO concentration 4.5%) were applied to the Deutch strain of *R. microplus*. Permethrin (analytical standard PESTANAL™, 91.8% sum of Cis, 30.3% + Trans, 61.5% isomers, product number 45614, Sigma‐AIdrich®, St. Louis, MO) 0.125% W/V (3.19 mM; MW = 391.29) in 1% MERO® in 5% DMSO was used as the positive control for mortality for this and all subsequent bioassays.

### 2.3 Small molecules

Small molecule antagonists of the tick kinin receptor SACC‐0412060, SACC‐0412062, SACC‐0412066, SACC‐0064443, SACC‐0428788, and SACC‐0039590 were previously identified and validated. For bioassays, these small molecules were ordered from commercial vendors (Table S1) and prepared as 100 mM stock solutions in 100% DMSO, except for SACC‐0064443 stock which was prepared at 50 mM because of its lower solubility. Small molecule solutions (10 mM) were prepared in 1% MERO® 5% DMSO, from which working concentrations of 1 mM were prepared in the same diluent. The larval immersion test was carried out in Eppendorf tubes with 1 mL of permethrin (as under section 2.2) or 1% MERO® in 5% DMSO as positive and negative controls, respectively, or 1 mM concentration of the small molecules.

### 2.4 Testing activity of solvents and small molecules on the *R. microplus* Deutch susceptible strain using the larval immersion test

To obtain larvae for the immersion tests, egg masses were placed in glass vials (1 cm diameter) closed with cotton and kept in a humid chamber (as explained below). Larvae used in all bioassays were 2 to 5 weeks old. The larval immersion test is standard for tick bioassays and was first described by Shaw in 1966 and modified by Sabatini *et al*.^39^, ^40^ For the larval immersion test, using the round end of a flat spatula, about 300–500 larvae of *R. microplus* Deutch susceptible strain were transferred into 1.5 mL Eppendorf tubes, each filled with 1 mL of the tested materials, either solvents or small molecules. The larvae were immersed for 10 min and incubated on a 3D rocker at room temperature. Larvae were transferred by pipette (using a 1 mL tip with the opening slightly cut) onto a filter paper to air‐dry. Finally, about 100 of the dry larvae were transferred using a spatula into each of the three packets prepared with Whatman chromatography paper number 1 (VWR), for which it had been cut into small sheets of 8 cm × 9 cm. The packets were closed with bulldog clips, hung from a rod and placed in a humid chamber (prepared using a closed glass aquarium containing a saturated solution of potassium nitrate (Spectrum Chemical, New Brunswick, NJ; CAS 7757‐79‐1) in tap water, to maintain the humidity) for 24 h at 28°C and 92% relative humidity. A solution of permethrin (as under section 2.2) was used as a positive control for larval bioassays, and negative controls were with 1% MERO® in 5% DMSO. After 24 h, the packets were opened onto a light box, and the dead and live larvae were counted under a magnification lens. Live larvae were considered those that could walk on the filter paper, which were aspirated as counted using a glass pipette connected to a vacuum pump.^41^ The larvae that could not walk after being touched with a thin brush were left on the filter paper and counted as dead. The percent mortality was calculated.

### 2.5 Lethal concentrations of SACC‐0039590 and SACC‐0428788 against *R. microplus* Deutch susceptible and pyrethroid‐resistant Arauquita strains using LIT

For estimation of the lethal concentrations (LC_50_, LC_90_, LC_99_) of SACC‐0039590, the following emulsions of 125, 85, 80, 70, 65, 62.5, 62, 60, 50, 40, 30 μM were prepared in 1% MERO® in 5% DMSO. For SACC‐0428788, concentrations of 1000, 500, 350, 250, 200, 175, 150, 125, 90, and 62.5 μM were prepared in 1% MERO® in 5% DMSO. For each concentration of the respective molecule series, 300–500 larvae of the Deutch‐susceptible and Arauquita‐resistant strains were immersed, as described above, in 1 mL of the respective solutions, for 10 min under slow agitation. Larvae were transferred with a pipette to dry on filter paper for a few seconds. Subsequently ~100 larvae of each concentration were placed with a spatula into respective three packets as mentioned under the LIT description. Positive (permethrin) and negative control packets prepared with 1% MERO® in 5% DMSO were included for each molecule concentration series.

### 2.6 Confirmation of pyrethroid resistance in the *R. microplus* Arauquita strain in comparison to the Deutch susceptible strain using the larval packet test (LPT)

To verify pyrethroid resistance in the Arauquita strain maintained in the CFTRL, permethrin crystals (92%) were dissolved in the diluent mix of trichloroethylene (two parts): highly refined low‐acidity olive oil (one part) (Sigma‐Aldrich), as described in previous studies.^42^, ^43^, ^44^ For testing the susceptible *R. microplus* Deutch strain, 10 mL of the highest concentration (0.1% permethrin w/v) stock was prepared and was serially diluted by transferring 7 mL from the highest to lowest concentrations, obtaining the following series: 0.07%, 0.049%, 0.034%, 0.024%, 0.016%, and 0.011%. For testing the resistant Arauquita resistant strain nine concentrations (W/V) of permethrin were prepared: 2.5%, 1.7%, 1.2%, 0.8%, 0.6%, 0.4%, 0.29%, 0.2%, 0.14%. About 0.7 mL of each concentration was applied to Whatman chromatography papers (no 1; 8 × 9 cm), and three papers were prepared for each treatment. The papers were hung to dry in a fume hood for 1 h (Fig. S1), then the packets were prepared and added with 100 larvae per packet. The packets were closed and immediately placed in a humid chamber as described in section 2.4. Dead and live larvae were counted after 24 h, as described in section 2.4.

### 2.7 Adult immersion test

Engorged female ticks (*n* = 120) of the Deutch strain that had dropped naturally from experimentally infested calves, were collected. Ticks were washed with tap water and dried on paper towels. Ticks were weighed and divided randomly into groups of 15 females, subdivided into three replicates of five ticks each per concentration. Treatment groups were as follows: distilled water; 1% MERO® in 5% DMSO (both as negative controls for mortality), positive control (permethrin 0.125% w/v), and five concentrations of SACC‐0039590 (1 mM, 0.65 mM, 0.5 mM, 0.325 mM, and 0.25 mM). Using 15 mL polypropylene tubes, females were immersed in groups of five in 5 mL of each concentration and placed for 10 min on a shaker with slow agitation, then removed, and dried on absorbent paper for 15–30 min.^45^ For each concentration five ticks were placed in each of three Petri‐dishes (100 mm x 15 mm) lined with qualitative filter paper (9 cm diameter) and fixed to it, with their ventral side up, using double‐sided tape. Females were incubated at 27–29 °C and 92% RH for 2 weeks to record reproductive variables. The eggs laid per concentration replicate (five ticks) were gathered and weighed. Eggs from the three replicates were placed in respective 10 mL‐glass vials, and the three vials were incubated at 27–29 °C and 92% RH for 3 weeks to estimate the percentage of hatching.

The equations below were used for estimation of the reproductive parameters.^46^, ^47^, ^48^


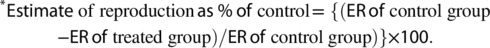




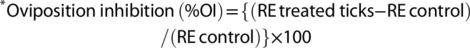




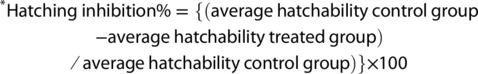




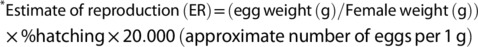




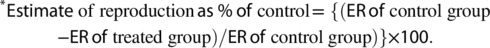




### 2.8 Statistical analysis

PoloSuite software version 2 (LeOra Software LLC, Parma, MO) was used for probit analyses to calculate LC_50_, LC_90_, and LC_99_ of SACC‐0039590, SACC‐0428788, and permethrin against the susceptible Deutch and Arauquita strains. Probit graphs were produced with SigmaPlot 15 software (Grafiti LLC, Palo Alto, CA). The histogram graphs were made using GraphPad Prism 10 (GraphPad Software, San Diego, CA).

Data of egg masses, reproductive efficiency index, and hatching percentage were analyzed using ANOVA followed by Tukey's multiple comparisons tests at a 95% confidence level using GraphPad Prism 10. For the estimation of reproduction reduction with respect to controls, one‐way ANOVA was followed by Dunnett's multiple comparison test (GraphPad).

## 3 RESULTS

### 3.1 Testing toxicity of the solvents and adjuvant combination

Using the larval immersion test, the solvents DMSO 5%; 0.5% MERO in 5% DMSO; 1% MERO in 5% DMSO, and 10% JEFFSOL in 5% DMSO were nontoxic to the susceptible *R. microplus* Deutch strain (Table 1). The mortality percentages ranged from 0% to 0.5% in the different treatments. There were no significant differences in mortality of the distilled water negative control group and the tested solvents DMSO 5%, 1% MERO in 5% DMSO, and 10% JEFFSOL in 5% DMSO. Permethrin 0.125% as positive control caused 100% mortality. The Tukey's multiple comparisons tests with 95% confidence level showed that permethrin was the only significantly different group. The novel solvent combinations 1% MERO in 5% DMSO, and 10% JEFFSOL in 5% DMSO were non‐toxic to the susceptible *R. microplus* Deutch strain (Table 1) and are suitable for bioassays. However, the treatment of 1% MERO in 5% DMSO was observed to best disperse the larvae in the tube during immersion, so it was adopted to evaluate the small molecules against *R. microplus* larvae.

**Table 1:** Evaluation of novel solvents' combinations on the susceptible *R. microplus* Deutch strain using the larval immersion test

Treatment	Larvae (n) per packet	Total No. of larvae	Mean mortality % ± S.E.^†^
Distilled water	179; 59; 112	350	0.00 ± 0.0^a^
DMSO 5%	134; 140; 255	529	0.50 ± 0.5^a^
0.5% MERO in 5% DMSO	127; 140; 140	407	0.24 ± 0.24^a^
1% MERO in 5% DMSO	200; 217; 148	565	0.17 ± 0.17^a^
10% JEFFSOL 1555 in 5% DMSO	192; 138; 209	539	0.00 ± 0.0^a^
Permethrin 0.125% (3.19 mM) in 1% MERO in 5% DMSO	100; 100; 100	300	100 ± 0.0^b^****

*Note*: Mortality percentages with the same letter in superscript are not significantly different (*P* ≥ 0.05). ****P ≤ 0.0001 (Tukey multiple comparison's tests after ANOVA).

### 3.2 Screening of small molecules for acaricidal activity using the larval immersion test (LIT)

In the LIT the mean mortality of the negative control (1% MERO in 5% DMSO) treatment of the Deutch susceptible strain was 0.17% after 24 h. The mean mortality percentages of the larvae tested with small molecules SACC‐0412060, SACC‐0412062, SACC‐0412066, SACC‐0064443, and SACC‐0039590 at 1 mM were below 4%, an insignificant acaricidal activity (Table 2). However, SACC‐0039590 and SACC‐0428788 showed 100%, and 73.65% mortality at 1 mM concentration. Permethrin (0.125%) resulted in 100% larvicidal activity against the Deutch susceptible strain. There was no significant difference between the mortality percentages of treated groups with SACC‐0039590 or Permethrin (3.19 mM), both dissolved in 1% MERO in 5% DMSO (Table 2).

**Table 2:** Efficacy of the small molecules against the *R. microplus* Deutch susceptible strain in the larval immersion test

Treatment	Concentration	Larvae (n) per packet	Total No. of larvae	Mean mortality % ± S.E.^†^
SACC‐0039590	1 mM	100, 100, 100	300	100 ± 0.0^c^
SACC‐0428788	1 mM	69, 114, 102	285	73.65 ± 8.9^b^
SACC‐0412060	1 mM	140, 120, 108	368	3.8 ± 1.4^a^
SACC‐0412062	1 mM	142, 136, 173	451	3.3 ± 1.9^a^
SACC‐0412066	1 mM	228, 124, 115	467	3.4 ± 2.4^a^
SACC‐0064443	1 mM	184, 91, 121	396	2.7 ± 1.99^a^
Negative Control (1% MERO in 5% DMSO)	1 mM	148, 217, 200	565	0.17 ± 0.17^a^
Permethrin 0.125%(w/v) in 1% MERO in 5% DMSO	3.19 mM	100, 100, 100	300	100 ± 0.0^c^

^†^ Mortality percentages with the same letter in superscript are not significantly different (*P ≥* 0.05).

### 3.3 Dose‐mortality responses of SACC‐0039590 and SACC‐0428788 against R. microplus susceptible Deutch strain using the larval immersion test (LIT)

**Figure 1:**
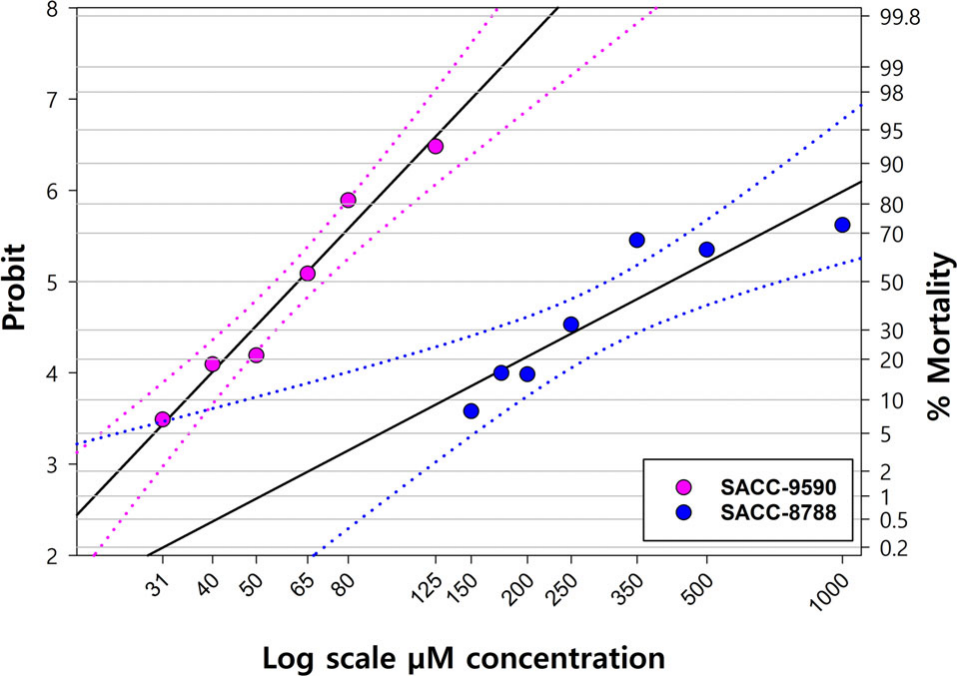
Probit analysis of *R. microplus* Deutch susceptible strain for SACC‐0039590 (LC_50_ = 60 μM) and SACC‐0428788 (LC_50_ = 450 μM) (larval immersion test).

The two molecules that showed high larvicidal activity in the screen at 1 mM were then assessed in concentration‐mortality bioassays with both the susceptible and resistant strains. Both molecules exhibited concentration‐dependent mortality against the Deutch strain (Fig. 1). For this strain the LC_50_, LC_90_, and LC_99_ of SACC‐0039590 were 60 μM, 100 μM, and 150 μM, respectively (Table 3). Lethal concentrations of SACC‐0428788 against the Deutch strain were 450 μM, 1501 μM, and 4001 μM for LC_50_, LC_90_, and LC_99_, respectively (Table 3). The lines were not parallel and not equal (*P* = 0.001) (Fig. 1). The molecule SACC‐0039590 was more potent than SACC‐0428788 against larvae of the Deutch strain.

**Table 3:** Lethal concentrations (LC_50_, LC_90_, LC_99_) of SACC‐0039590 and SACC‐0428788 against *R. microplus* susceptible Deutch strains using the larval immersion test

Small molecule	No. of Larvae	Chi Square	DF	Slope ± SE	LC_50_ μM (95% CI)	LC_90_ μM (95% CI)	LC_99_ μM (95% CI)
SACC‐0039590	4228	146.08	9	6.59 ± 0.4	60 (54–71)	100 (88–159)	150 (112–359)
SACC‐0428788	1645	86.4	4	2.45 ± 0.16	450 (222.9–1288)	1501 (732.8–129 444)	4001 (1354‐ >10^6^)

### 3.4 Confirmation of pyrethroid resistance in the *R. microplus* Arauquita strain in comparison to the Deutch susceptible strain using the larval packet test (LPT)

**Figure 2:**
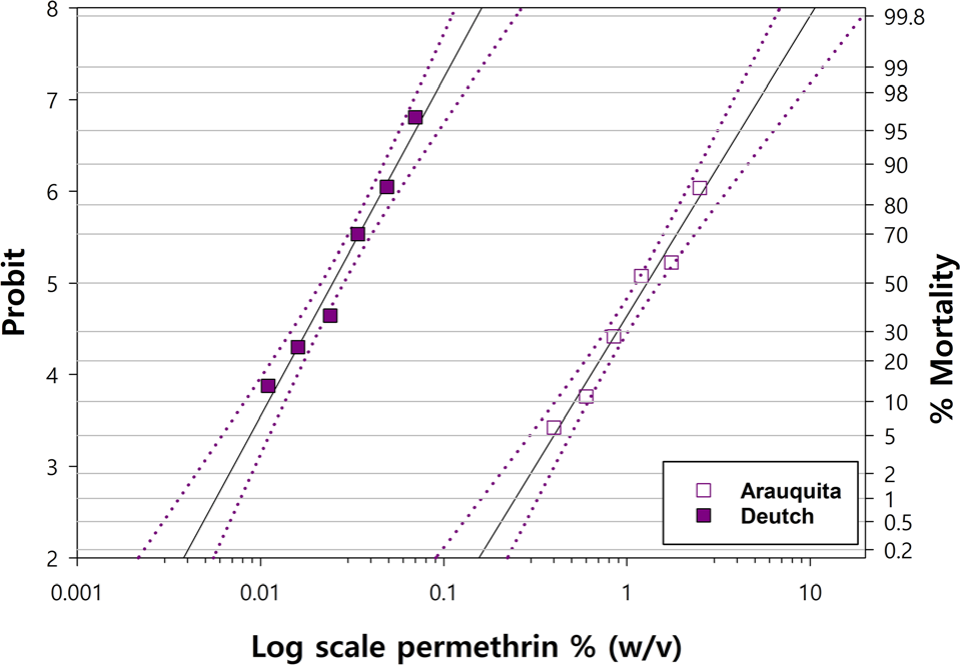
Probit analysis of *R. microplus* Deutch susceptible strain (LC_50_ = 0.03% W/V) and Arauquita resistant strain for permethrin (LC_50_ = 1.38% W/V) using the larval packet test.

The Arauquita strain had been last subjected to pyrethroid challenge in 2018 at the CFTRL, therefore, it was necessary to verify its current level of pyrethroid resistance. Concentration‐mortality responses were obtained for permethrin against the Deutch susceptible and Arauquita resistant strains (Fig. 2). The highest concentration of 0.1% w/v achieved 100% larval mortality for the Deutch strain. The lethal concentrations and the probit analysis results are shown in Table 4, LC_50_ = 0.03, LC_90_ = 0.06, LC_99_ = 0.09% W/V. The *R. microplus* Arauquita strain was resistant with lethal concentrations of 1.38, 3.06, and 5.87% w/v permethrin for the LC_50_, LC_90_, and LC_99_, respectively. The resistance ratio for the LC_50_ (RR_50_) of the Arauquita strain to the Deutch strain was 46 (C.I. 41.8–48.6) (Table 4 and Fig. 2).

**Table 4:** Lethal concentrations (LC_50_, LC_90_, LC_99_) of permethrin % (w/v) against *R. microplus* susceptible Deutch and resistant Arauquita strains using the larval packet test

Strain	No. of Larvae	Chi Square	DF	Slope ± SE	LC_50_% (95% CI)	LC_90_% (95% CI)	LC_99_% (95% CI)	Resistance ratio LC_50_ (95% CI)
Deutch susceptible	2172	10.29	4	4.9 ± 0.3	0.03 (0.026–0.034)	0.06 (0.049–0.066)	0.09 (0.074–0.125)	46 (41.8–48.6)
Arauquita pyrethroid‐resistant	4411	29.3	3	3.69 ± 0.17	1.38 (1.068–1.77)	3.06 (2.25–6.15)	5.87 (3.63–19.32)	

### 3.5 Efficacy of SACC‐0039590 and SACC‐0428788 against the resistant Arauquita strain

**Figure 3:**
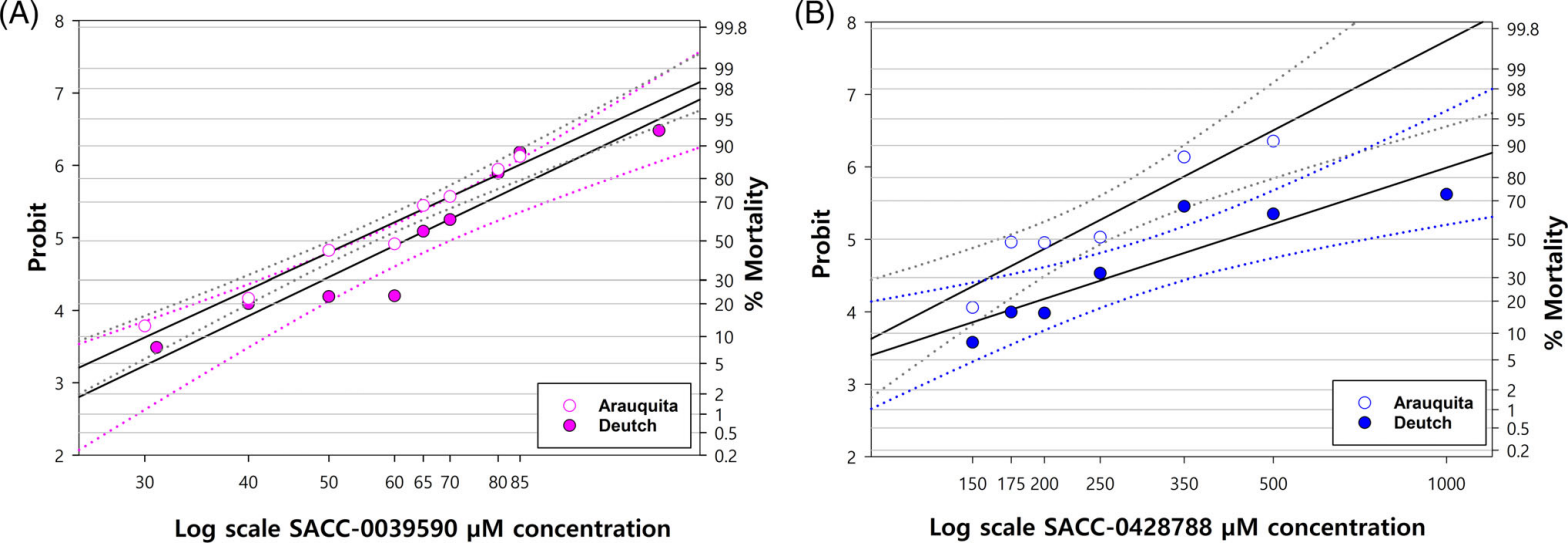
Larval immersion test. (A) Probit analysis of *R. microplus* Arauquita resistant strain (LC_50_ = 60 μM) and Deutch susceptible strain for SACC‐0039590 (LC_50_ = 60 μM) using the larval immersion test. (B) Probit analysis of *R. microplus* Arauquita strain (LC_50_ = 237.48 μM) and Deutch susceptible strain (LC_50_ = 450 μM) for SACC‐0428788 using the larval immersion test.

Both molecules SACC‐0039590 and SACC‐0428788 were simultaneously tested against the Arauquita pyrethroid‐resistant strain and the Deutch strain, but for clarity the results are shown individually in comparison to the susceptible strain in Fig. 3. The LC_50_, LC_90_, and LC_99_ of SACC‐0039590 against the resistant Arauquita strain were 60 μM, 90 μM, and 130 μM, respectively (Table 5). The probit lines were statistically similar and there were no significant differences between the slopes of the two strains (*P* = 0.7) (Fig. 3A, Table 5). This indicates that SACC‐0039590 is equally effective to control susceptible and pyrethroid‐resistant larvae of the Arauquita strain.

**Table 5:** Lethal concentrations (LC_50_, LC_90_, LC_99_) of SACC‐0039590 and SACC‐0428788 against the resistant Arauquita strains using the larval immersion test

Small molecule	No. of Larvae	Chi Square	DF	Slope ± SE	LC_50_ μM (95% CI)	LC_90_ μM (95% CI)	LC_99_ μM (95% CI)
SACC‐0039590	4380	38.158	9	6.58 ± 0.31	60 (54–61)	90 (85–100)	130 (116–159)
SACC‐0428788	1829	30	3	4.18 ± 0.31	237.48 (92.7–324)	480.6 (347.5–2303.8)	853.81 (514.3–22 624.8)

For SACC‐0428788 the lethal concentrations were higher than for SACC‐0039590 against the Arauquita strain, with LC_50_ = 237.5 μM, LC_90_ = 480.6 μM, and LC_99_ = 853.8 μM, indicating lesser potency (Fig. 3B, Table 5). There was a significant difference (*P* = 0.01) between the slopes of probit lines between the Deutch and Arauquita strain, but there was no significant statistical difference in the mortality percentages for both strains (Fig. 3B). Therefore, SACC‐0039590 was more potent than SACC‐0428788 but there is no difference in the mortality between strains for both molecules.

### 3.6 Effect of SACC‐0039590 on Deutch susceptible strain engorged females using the adult immersion test (AIT)

**Figure 4:**
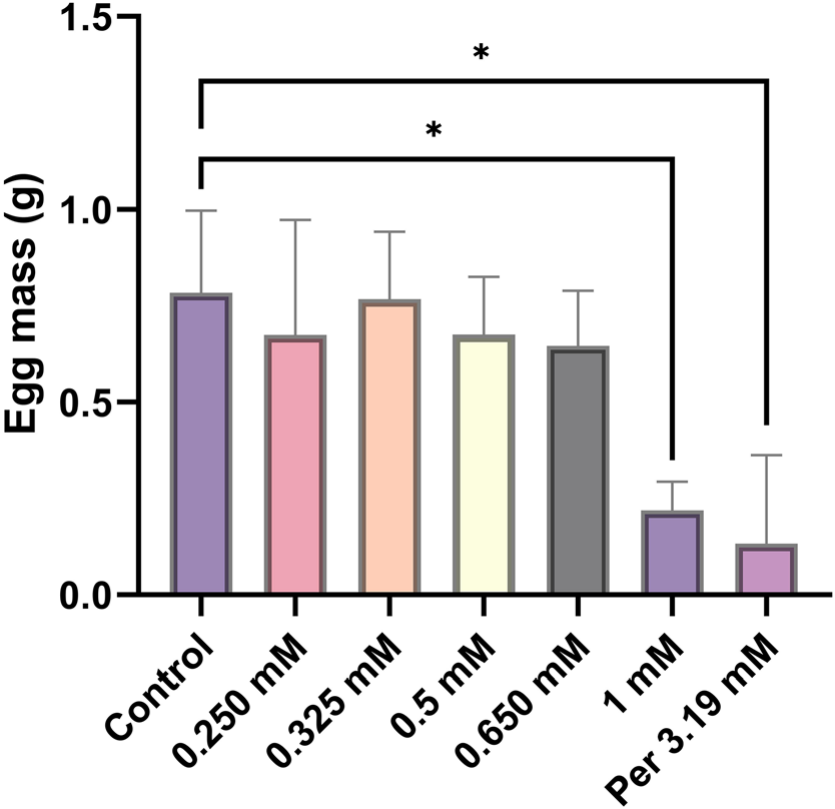
Egg mass weight (g) of Deutch females treated with different concentrations of SACC‐0039590 using the adult immersion test (mean ± SE of three replicates). SACC‐0039590 only at 1 mM significantly (*P* ≤ 0.05) reduced the egg mass of the treated females in comparison to the control (1% MERO in 5% DMSO). The permethrin 0.125% (3.19 mM) treated group showed a similar significant (*P* < 0.05) reduction in the egg mass.

**Figure 5:**
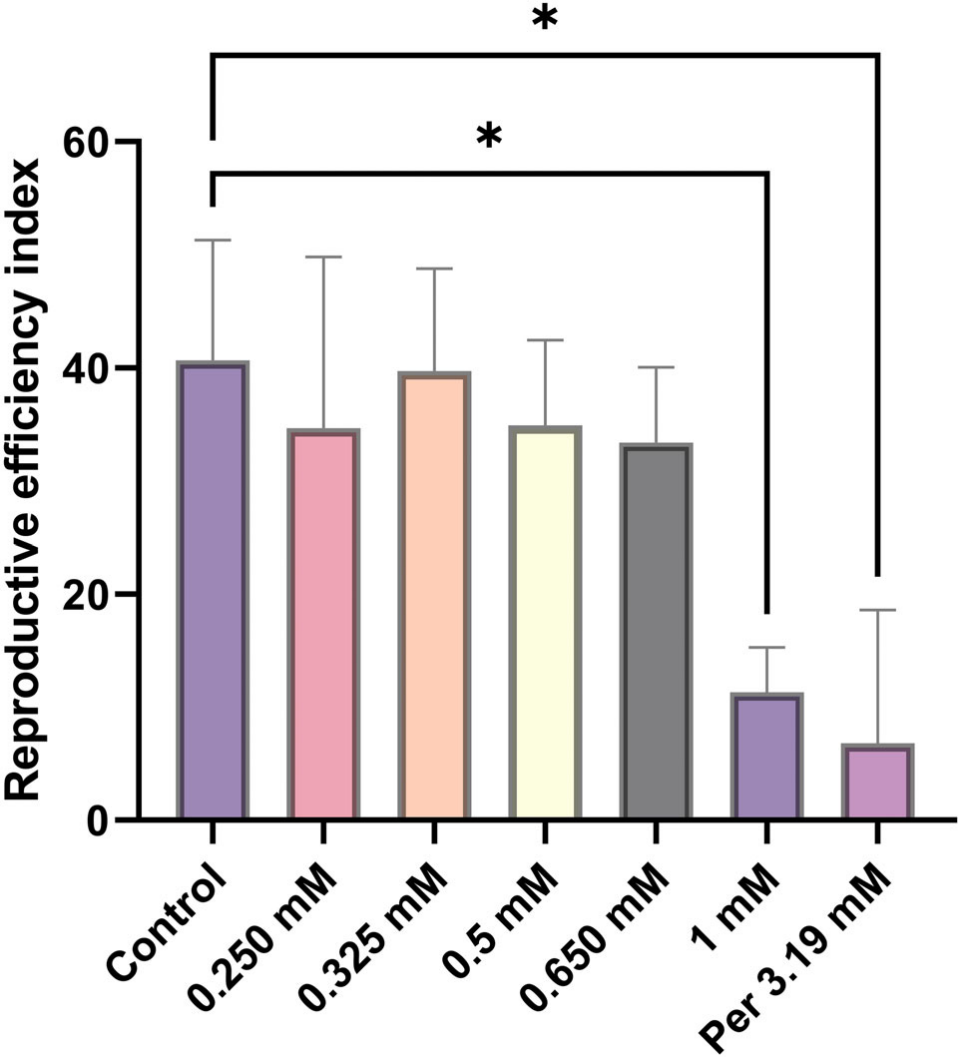
Reproductive efficiency index (REI) of Deutch females after immersion in different concentrations of SACC‐0039590 (mean ± SE of three replicates). SACC‐0039590 only at 1 mM significantly (*P ≤* 0. 05) reduced the REI in comparison to the control (1% MERO in 5% DMSO). The permethrin 0.125% (3.19 mM) treated group showed a similar significant (*P* < 0.05) reduction in REI.

Statistical analysis of the AIT data using ANOVA showed there was a significant difference (*P* ≤ 0.05) in the egg mass production and reproductive efficiency index among the different *R*. *microplus* Deutch treated groups (Table 6, Fig. S2). Multiple mean comparisons using the Tukey's test showed that SACC‐0039590 at 1 mM concentration significantly reduced (*P* ≤ 0.05) the mean egg mass (0.22 g) in comparison to the control 1% MERO in 5% DMSO (0.78 g) (Fig. 4, Table 6). Moreover, a significant reduction (*P* ≤ 0.05) of the reproduction efficiency index % was observed with SACC‐0039590 at 1 mM in comparison to the control 1% MERO in 5% DMSO (11% *vs* 41%) (Fig. 5, Table 6). Similarly, at 1 mM, the inhibition of oviposition percentage was 76.5% with respect to the same control. SACC‐0039590 at 1 mM, 0.650 mM, 0.5 mM significantly reduced hatchability (*P* ≤ 0.05) (Table 6). On the contrary, concentrations of SACC‐0039590 less than 0.5 mM did not induce significant effects on the reproductive parameters in comparison to the control. Permethrin 0.125% showed significant reduction (*P* ≤ 0.05) of egg mass deposition (0.13 g), and egg production index % (6%) in comparison to the control 1% MERO in 5% DMSO. Permethrin 0.125% induced 85.86% inhibition of oviposition in comparison with the control. Permethrin 0.125% did not reduce significantly (*P* ≥ 0.05) the hatchability (75%) and showed 86.73% reduction of the estimated reproduction (Table 6).

**Table 6:** Effect of different concentrations of SACC‐0039590 on reproductive parameters of immersed engorged Deutch strain females (data are the mean of three replicates consisting of 5 ticks each)

Treatment	Number of ticks per replicate	Female weight (g) per replicate (Mean ± SE)	No ticks not laying eggs per replicate (Mean ± SE)	Egg mass (g) per replicate (Mean ± SE)	Reproductive Efficiency Index per replicate (Mean ± SE)	Inhibition of oviposition %	Hatching %	Hatching inhibition %	Reproduction reduction (% of control ER)
Distilled water	5	1.94 ± 0.02	0.33 ± 0.33	0.93 ± 0.11^a^	48 ± 5. 5^a^	0	95 ± 0.0 ^a^	0	0 ^a^
1% MERO in 5% DMSO (Control)	5	1.92 ± 0.01	0.66 ± 0.33	0.78 ± 0.12^a^	41 ± 6^a^	15.54 ± 12.77^a^	95 ± 0.0 ^a^	0	0 ^a^
SACC‐9590 (1 mM)	5	1.95 ± 0.01	3.0 ± 0.57	0.22 ± 0.04^b^	11.3 ± 2.3b	76.50 ± 4.80^b^	58.33^c^ ± 8.33	38.59	79.29 ± 5.06^b^
SACC‐9590 (0.650 mM)	5	1.93 ± 0.25	0.33 ± 0.33	0.65 ± 0.08^a^	33.4 ± 3.8^a^	30.69 ± 8.01^a^	60 ± 10^c^	36.84	49.86 ± 4.2^a^
SACC‐9590 (0.5 mM)	5	1.93 ± 0.008	0.66 ± 0.33	0.68 ± 0.09^a^	35 ± 4.3^a^	27.51 ± 9.03^a^	66.67 ± 8.33^b^	29.82	40.66 ± 9^a^
SACC‐9590 (0.325 mM)	5	1.93 ± 0.017	0.33 ± 0.33	0.77 ± 0.1^a^	40 ± 5.24^a^	17.55 ± 10.89^a^	80 ± 5^a^	15.79	19.29 ± 6.4^a^
SACC‐9590 (0.250 mM)	5	1.94 ± 0.01	0.66 ± 0.66	0.67 ± 0.17^a^	35 ± 8.7^a^	28.04 ± 18.17^a^	75 ± 0^a^	21.05	32.7 ± 16.96^a^
Permethrin 0.125% (3.19 mM)	5	1.94 ± 0.005	3.3 ± 1.7	0.13 ± 0.13^b^	6.8 ± 6.8^b^	85.86 ± 14.14^b^	75^a^	21.05	86.73 ± 13.19^b^

*Note*: Different letters in superscript indicate significant differences among concentrations (*P* ≤ 0.05).

Abbreviations: ER, estimated reproduction.

## 4 DISCUSSION

Populations of *Rhipicephalus microplus* negatively affect livestock worldwide because they transmit deadly pathogens and reduce animal productivity. Focusing on G protein‐coupled receptors (GPCRs) as novel targets is a recognized promising approach for tick control.^18^, ^20^ For example, the commercial acaricide Amitraz, a formamidine, activates a tyramine/octopamine receptor.^19^, ^20^, ^21^, ^22^ To continue the search for other promising GPCR as targets, here we investigated and documented the acaricidal activity of two small molecules that are weak antagonists of the tick recombinant kinin receptor (IC_50_ > 25 μM),^49^ a neuropeptide GPCR, on *R. microplus* acaricide susceptible and resistant ticks. We have focused our screening efforts on the tick kinin receptor for selectivity, because the kinin receptor is not present in vertebrates.^20^ The first challenge of this study was to search for suitable solvents and adjuvants to enhance the penetration of small synthetic molecules through the hard tick cuticle. Dimethyl sulfoxide (DMSO) is a universal solvent miscible with water and many organic solvents, thus, it is commonly used to dissolve both polar and nonpolar compounds.^50^ DMSO can disrupt the H‐bonds of chitin that builds the cuticle of insects.^50^, ^51^, ^52^ DMSO is a penetration enhancer, its effect on cell membranes ranges from thinning to destroying the bilayer structure and pore formation, depending on the concentration.^53^

The rationale for including JEFFSOL in our initial testing was that based on the Huntsman technical bulletin^54^ this commercial propylene carbonate is a safe solvent (minimal risk classification) and is approved for agricultural pesticide formulations. Specifically, applications are in veterinary topical formulations and as a component of tank adjuvants; it has a relative vapor pressure of 0.03 Pa when that of water is considered as 1.^55^ Although the safety of 10% JEFFSOL in DMSO 5% against *R. microplus* was recorded in this work, this combination could not disperse the tick larvae in the tube. Consequently, this combination was not selected to test the small molecules.

MERO was investigated because it reduces the surface tension and improves the efficacy of insecticides against mosquitoes.^56^ The combination of MERO‐acetone was reported in many studies as nontoxic for mosquitoes and increased the potency of neonicotinoid insecticides^56^, ^57^, ^58^; however acetone is toxic to ticks so we discarded this as a suitable pairing.^59^ We found that 1% MERO in DMSO 5%, a novel solvent combination, was nontoxic on *R. microplus* larvae of both Deutch susceptible and Arauquita resistant tick strains, as there was not a significant difference in mortality between the solvent‐treated and the water‐treated larvae. Moreover, we found that this solvent combination was nontoxic when *R. microplus* larvae were immersed in it. MERO is a rapeseed fatty acid esters formulation, which at 800 ppm was nontoxic to *Anopheles* mosquitoes.^60^ The emulsifying properties of MERO as soap, increased the solubility of insecticides, preventing their crystallization.^56^, ^61^ Similarly, soap at a concentration of less than 1% is nontoxic to mosquitoes.^62^ MERO at 1% concentration was effective with clothianidin 150 μg/mL exhibiting 100% mortality against *An. gambiae*.^56^ MERO enhances the activity of clothianidin, acetamiprid and imidacloprid against mosquitoes at low concentrations.^57^, ^60^ MERO is a nonpolar molecule with a long hydrocarbon chain. It has limited capacity to overcome charged molecular interactions or dissolve polar substances, but it is a good solvent for nonpolar molecules.^63^ MERO has a lower relative volatility (vapor pressure 5 × 10^−6^ Pa) than propylene carbonate and water.^55^

One benefit of MERO was the observation that during the LIT the larvae did not aggregate while immersed in 1% MERO in 5% DMSO in comparison to DMSO 5%, and 10% JEFFSOL in 5% DMSO. In the case of 5% DMSO alone, the hydrophobic larvae were stuck to the tube and were difficult to extract at the end of the test.

Acaricide resistance for the different pesticide classes has been reported in many countries.^8^, ^9^, ^64^, ^65^, ^66^, ^67^, ^68^ In the current study, the Deutch acaricide susceptible strain was tested with permethrin and exhibited an LC_99_ of 0.09% w/v. Previously, the reported the LC_99_ for permethrin was 0.125% w/v (1250 ppm; 3.19 mM) for the Deutch stain, and the resistance discriminating dose (DD) was considered twice the value of the LC_99_.^10^, ^43^ For this reason, the 0.125% w/v of permethrin was used for comparison to the small molecules. Although the resistance status of *R. microplus* Arauquita strain was documented previously, it was important to confirm its current permethrin resistance to validate the activity of the small molecules on a well‐known resistant strain. The highest concentration of permethrin at 0.25% w/v, the putative discriminating dose for resistance, killed only 4.6% of the *R. microplus* Arauquita resistant strain, confirming its resistant status. In the current study, the resistance ratio RR_50_ of Arauquita strain compared to the Deutch strain was 46 using the standard larval packet test.

Similarly, in another report, the deltamethrin resistance ratio (RR_50_) of the Arauquita strain was 241 in a comparison with the Deutch strain using the larval immersion test.^15^ In addition, this strain showed resistance for organophosphorus compounds, as chlorpyrifos at 312 ppm showed only 64% mortality.^15^ Similar studies showed that both cypermethrin and deltamethrin completely lost their efficacy against *R. microplus* in many farms in Colombia.^69^ Single nucleotide polymorphism (SNP) analyses of the voltage‐gated sodium channel gene of 27 tick females of *R. microplus* Arauquita strain identified 10/27 homozygous resistant mutants, 13/27 heterozygous mutants, and 4/27 were homozygous susceptible.^15^ In contrast, the same *R. microplus* Arauquita strain exhibited ivermectin susceptibility in the LIT.^15^

It is noteworthy that both small molecules SACC‐0039590 and SACC‐0428788 showed larvicidal activity against both Deutch susceptible and Arauquita resistant strains without significant differences between strains for each molecule (Fig. 3), indicating that the mode of action of these structural analogs that differ by the presence of a methyl group in SACC‐0039590 (Table S1), is likely not the voltage gated sodium channel. The estimated lethal concentrations of SACC‐0039590 against the susceptible Deutch and Arauquita strains were similar LC_50_ = 60 μM (Fig. 3A).

We previously determined that SACC‐0039590 and SACC‐0428788 were non‐cytotoxic to human dermal fibroblasts (HDF) and when SACC‐0039590 was applied at 20 μM–inhibited <10% of the fluorescence signal elicited by the recombinant cells expressing the kinin receptor after stimulated by a kinin agonist.^35^

The small molecule SACC‐0039590 is N4‐(4‐chlorophenethyl)‐5‐methylthieno[2,3‐d] pyrimidin‐4‐amine. This thienopyridine derivative was cytotoxic to murine leukemia cells.^70^ The anticancer activities of methylthionine[2,3‐d] pyrimidin‐4‐amine have been documented in many publications.^71^, ^72^ Moreover, the anti‐inflammatory, anti‐fungal, and antimicrobial activities of this molecule have been documented.^70^, ^73^, ^74^, ^75^ Activity of the thieno[2,3‐d]pyrimidin‐4‐amine analogues as kinases inhibitors was reported.^76^, ^77^ Moreover, thieno[2,3‐d]pyrimidine showed anthelmintic activity against adult Indian earthworms^78^ and the 2,4‐Diaminothieno[3,2‐d] pyrimidines, showed anthelmintic activity against *Trichuris trichiurid* adult worm and egg stages.^79^ The molecule SACC‐0428788 is a structural analog of SACC‐0039590 and showed larvicidal activity against both Deutch and Arauquita *R. microplus* larvae, however, with less potency than SACC‐0039590. The LC_50_ values of SACC‐0428788 were 450 μM (Table 3), and 237.48 μM (Table 5) for the Deutch and Arauquita *R. microplus* larvae, respectively, therefore, this molecule is roughly less potent than SACC‐0039590 by factors of 7.5 for the Deutch strain (450 μM / 60 μM; Tables 3) and of ~4 for the resistant Arauquita strain (237 μM/ 60 μM) (Table 5). Both molecules contain a thieno[2,3]pyrimidine scaffold, a chlorophenyl group and an ethylamine linker, while only SACC‐0039590 features a 5‐methyl group in the thieno[2,3]pyrimidine core.

Although the small molecules SACC‐0412060, SACC‐0412062, SACC‐0412066, and SACC‐0064443 showed potent antagonistic activity on the recombinant kinin receptor from *R. microplus*,^35^ they were not acaricidal in the current study. In *Aedes aegypti*, kinins have a regulatory role in hindgut muscle contraction^80^ and the receptor was immunolocalized in the female hindgut.^28^, ^81^ The first three of these antagonists (SACC‐0412060, −2062 and − 2066) significantly reduced the myostimulatory effect of the potent kinin agonist analog 1728 on the hindgut of *Aedes aegypti* mosquitoes.^35^ The fourth was not tested because it was not commercially available. In *Drosophila* the kinin receptor has a role in the larval tracheal air‐filling.^82^ In *R. microplus*, silencing of the tick kinin receptor decreased the reproductive fitness, lowered the egg hatching percentage, and discolored the midgut.^29^ Insect kinins regulate many vital biological activities in insects.^20^, ^25^ In *Aedes aegypti*, kinins have a regulatory role in diuresis and the receptor directly regulates sugar taste perception in sensory neurons.^28^, ^81^ Insects kinins regulate feeding, hindgut contraction, and the release of digestive enzymes in the gut.^31^

Immersion of the engorged female ticks in SACC‐0039590 at concentrations 0.5 to 1 mM reduced the reproductive parameters. There was a significant decrease in the egg mass deposition, and hatchability. Similarly, silencing LKR in *R. microplus* ticks affected the feeding and decreased the reproductive efficiency index and hatching percentage.^29^ In the case of serum fed ticks, light guts were observed and resulted in stopping the embryogenesis.^83^ Silencing of LKR likely resulted in reduced midgut peristalsis,^34^ consequently, delaying the egg production, and decreasing the egg mass weight.^29^ The effects of this weak kinin receptor antagonist on engorged females are similar to the phenotype observed when silencing the kinin receptor in females of *R. microplus*.^29^

In conclusion, in the current study the novel non‐toxic solvent combinations of 1% MERO in DMSO 5% was useful for testing candidate small molecule acaricides. SACC‐0039590 and its analog SACC‐0428788. Both molecules showed larvicidal activity against both susceptible and resistant *R. microplus* larvae. On the engorged female ticks, SACC‐0039590 reduced the egg mass weight, reproductive efficiency index, and egg hatching percentage.

## AUTHOR CONTRIBUTIONS

WMA: methodology, investigation, formal analysis, visualization, writing original draft, and editing the final manuscript. KHL: supervision (DK, JV), resources, methodology, administration, and editing the final manuscript. PVP: conceptualization, investigation, design, resources, project administration, funding acquisition, supervision (WMA), writing and editing the final manuscript.

## CONFLICT OF INTEREST STATEMENT

A United States patent application Pub. No.: US 2024/0251785 A1 has been filed Pub. Date: Aug. 1, 2024, Patricia V. Pietrantonio, Caixing Xiong and Dwight D. Baker. Small molecule antagonists and agonists of arthropod kinin receptors for pest control. A Provisional Patent Application is being prepared P14705US00. P. V. Pietrantonio, James C. Sacchettini, C. Xiong, and B. M. Henriques‐Santos. Small molecules as acaricidal kinin receptor antagonists or mosquitocidal agents.

## FUNDING SOURCES

This project was supported by the Agriculture and Food Research Initiative Competitive Grant no. 2022‐67015‐36336 from the USDA National Institute of Food and Agriculture, and partially by the Animal Health and Disease Research Capacity Funding (FY’23) grant (USDA‐NIFA Accession # 7004322), and the Texas A&M AgriLife Research Insect Vector Diseases Seed Grant Program (FY ’22‐’23 and FY ‘24‐‘25). NIFA also supports PVP program through Research Capacity Funds, Hatch project TX 9206 (Accession # 7006180). These sponsors were not involved in the study design, collection, analysis, or interpretation of data.

## Supporting information


**Table S1.** The small molecules evaluated, vendors, molecular weight (M.W.), vendor identification number (ID), inhibitor concentration 50 on the tick kinin receptor (IC_50_), and their molecular structure.
**Fig. S1.** Photograph of impregnated papers with the different permethrin concentrations for larval packets (left), and larval packets loaded with the larvae (right).
**Fig. S2.** Photographs of egg mass deposition of *R. microplus* Deutch engorged female ticks 14 days post immersion.

## Data Availability

Most of the data are in the manuscript or supporting information. The data that support the findings of this study are available from the corresponding author upon reasonable request.
