# Supplementary material for: Acaricidal activity of small molecule antagonists of the tick kinin receptor against Rhipicephalus microplus acaricide‐susceptible and resistant strains
Source: Pest Manag Sci. 2025 Oct 25;82(2):1618–28. doi: 10.1002/ps.70309 (PMC12790644; doi:10.1002/ps.70309)
Supplement: Supplementary file 1 — Table S1. The small molecules evaluated, vendors, molecular weight (M.W.), vendor identification number (ID), inhibitor concentration 50 on the tick kinin receptor (IC50), and their molecular structure. Fig. S1. Photograph of impregnated papers with the different permethrin concentrations for larval packets (left), and larval packets loaded with the larvae (right). Fig. S2. Photographs of egg mass deposition of R. microplus Deutch engorged female ticks 14 days post immersion. [file PS-82-1618-s001.docx]

Table S1.

The small molecules evaluated, vendors, molecular weight (M.W.), molecule vendor identification number (ID), inhibitor concentration 50 on the tick kinin receptor (IC_50_), and their molecular structure.

| Small molecule | Vendor | M.W | ID | IC_50_ on recombinant tick kinin receptor (BMLK3)  (µM ) | Structure |
| --- | --- | --- | --- | --- | --- |
| 1-  SACC-0064443 | AKos (Germany) | 410.91 | AKOS000532038 | 0.67 |   CAS-303028-29-7 |
| 2-  SACC-0412062^*^ | Chembridge (USA) | 440.55 | MCULE-6922861110 | 1.18 |   CAS-301222-97-9 |
| 3-  SACC-0412060^*^ | Mcule (USA) | 376.46 | MCULE-6534248485 | 1.89 |   CAS-300818-66-0 |
| 4-  SACC-0412066^*^ | Chembridge (USA) | 450.54 | MCULE-7946993717 | 3.22 |   CAS-2862024-71-1 |
| 5-  SACC-0039590 | MolPort (Latvia)  ChemSpace (USA) / | 303.81 | MolPort-001-806-190  CSMS00104487576 | ˃ 25 | 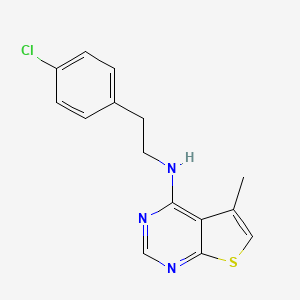  CAS-228407-20-3 |
| 6-  SACC-0428788** | Enamine (USA) | 289.78 | Z31244551 | ˃ 25 | 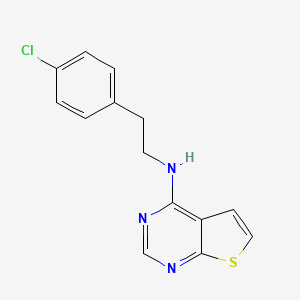  CAS-138040-46-7 |

Footnote: The IC_50_ values were obtained from Xiong et al., 2021

* From 2 to 4: SACC-0412062, SACC-0412060, SACC-0412066 are structural analogs of SACC-0064443 (Molecule 1), a small molecule in the Texas AgriLife Research small molecule chemical library in the laboratory of Professor James Sacchettini and original antagonist hit (Xiong et al., 2021). These data were obtained from the supplementary material of the published data (Xiong et al., 2021). ** SACC-0428788 (unpublished data) is a structural analog of SACC-0039590, which was originally identified as antagonist in the high-throughput screen.


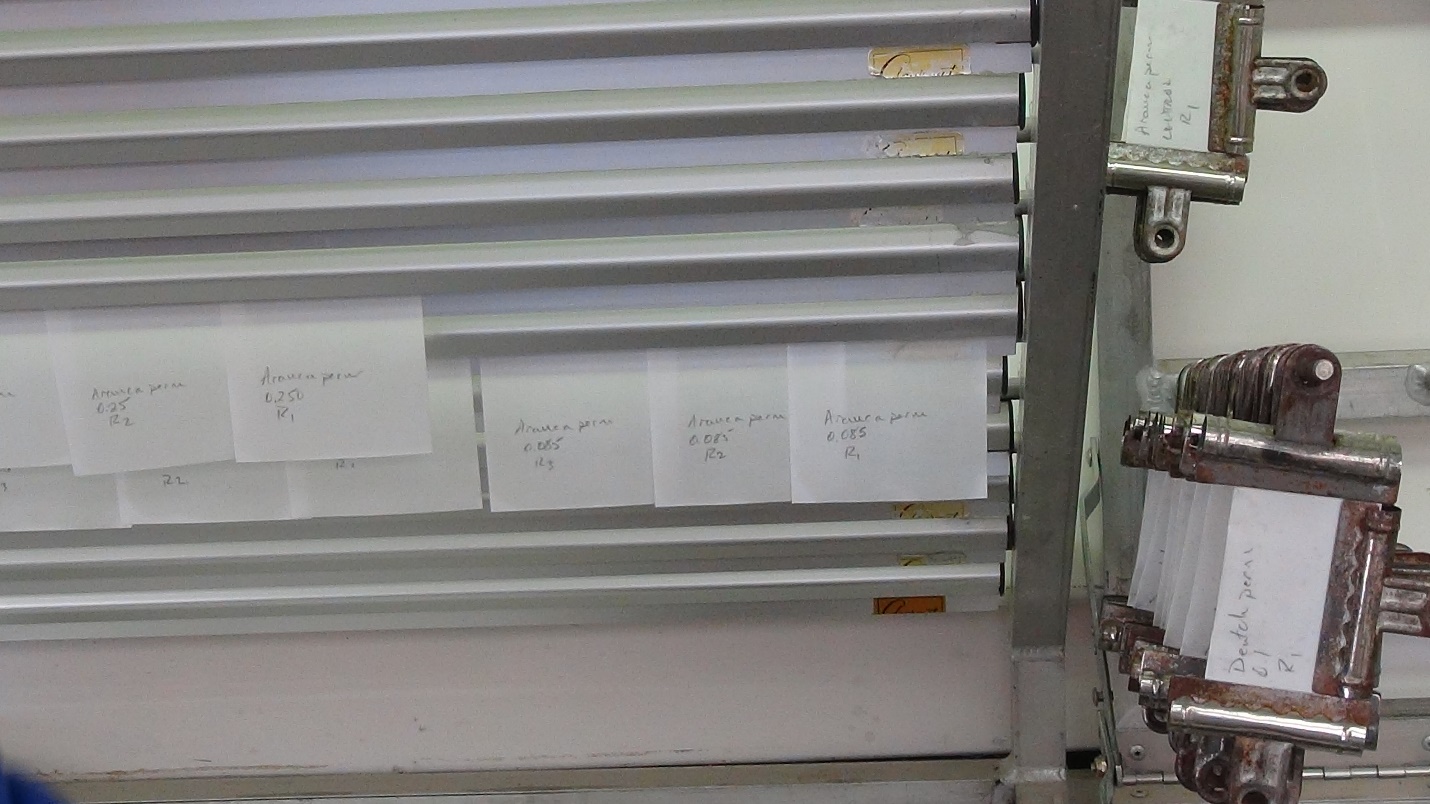


Figure S.1

Photograph of impregnated papers with the different permethrin concentrations for larval packets (left), and larval packets loaded with the larvae (right).

Figure S.2

Photographs of egg mass deposition of *R. microplus* Deutch engorged female ticks14 days post immersion. A) Distilled water group. B) 1% MERO in 5% DMSO treated group. C) 1 mM SACC-0039590 treated group. SACC-0039590 only at 1 mM significantly (*P ≤ 0.05*) reduced the egg mass of the treated females in a comparison to the control (1% Mero in 5% DMSO). D) Permethrin 0.125% treated group showed a similar significant (*P < 0.05*) reduction in the egg mass.

D


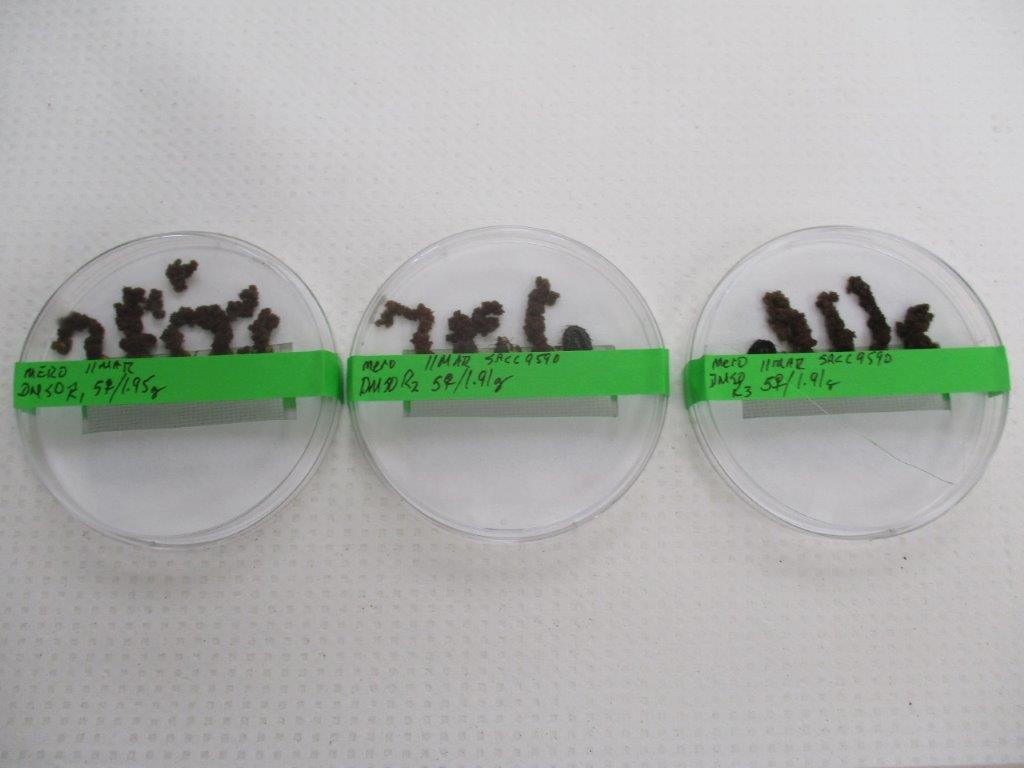

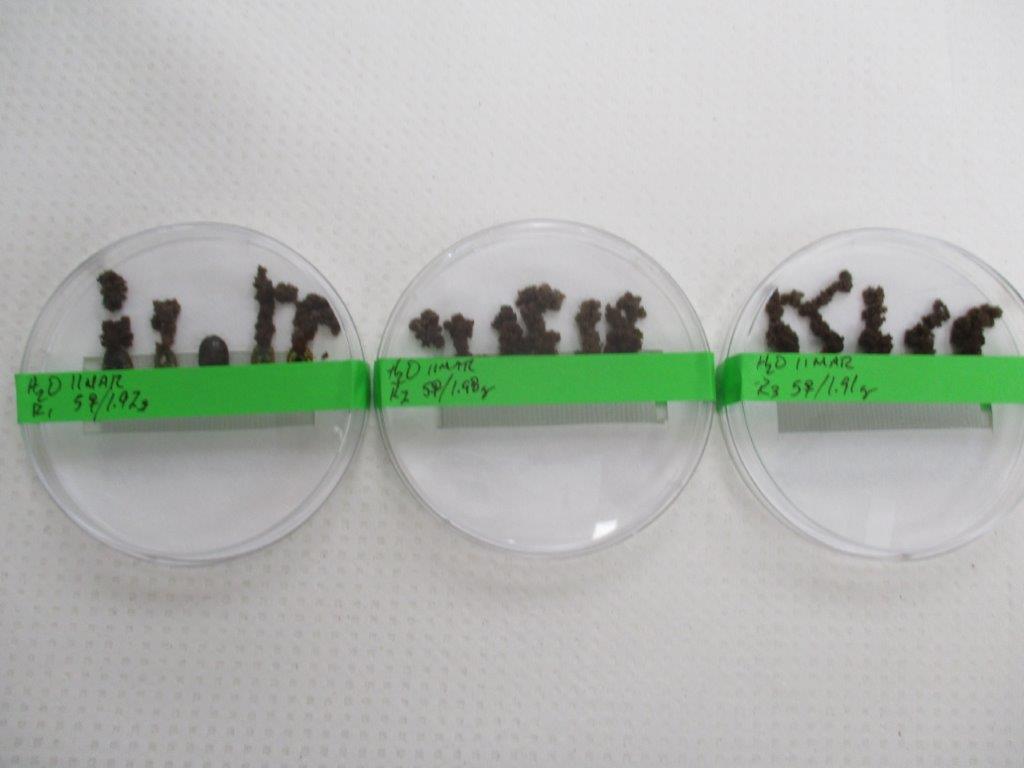

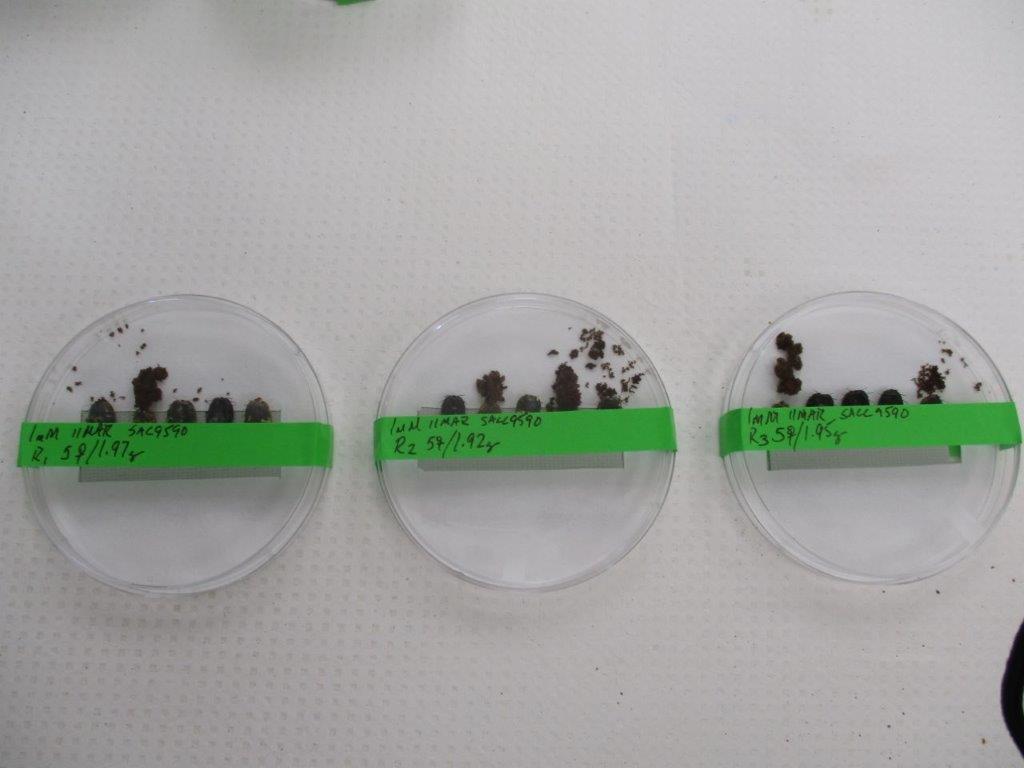

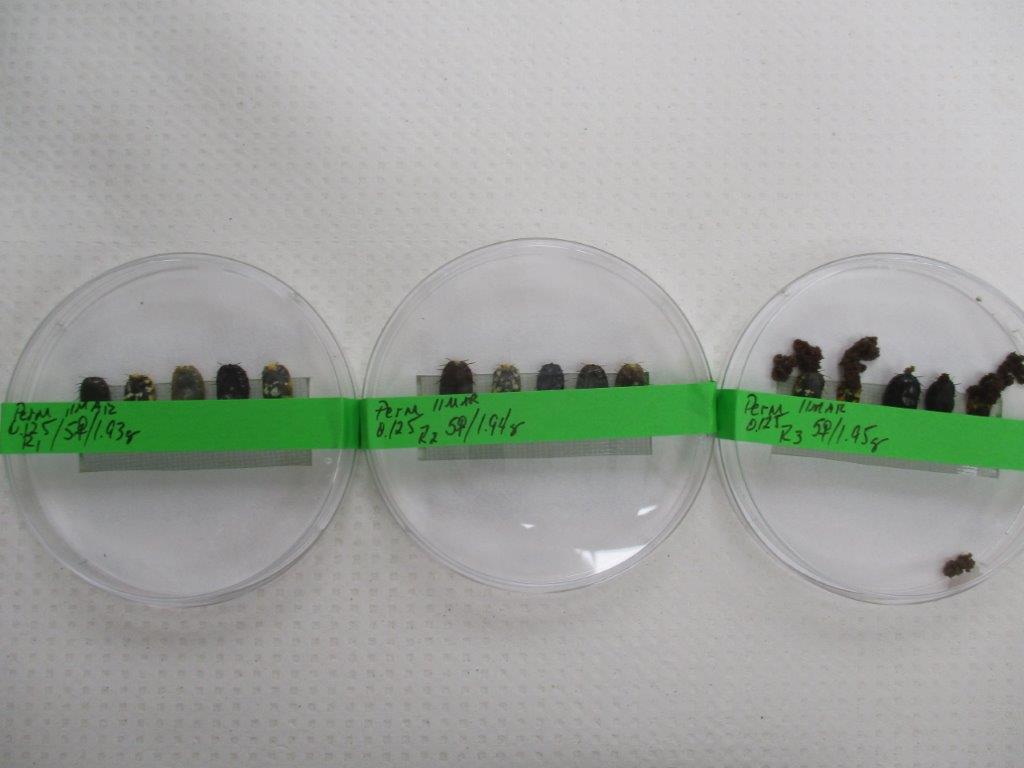


C

B

A
